# Novel diaminoguanidine functionalized cellulose: synthesis, characterization, adsorption characteristics and application for ICP-AES determination of copper(II), mercury(II), lead(II) and cadmium(II) from aqueous solutions

**DOI:** 10.1186/s13065-022-00857-3

**Published:** 2022-08-30

**Authors:** Magda A. Akl, Mohammed A. Hashem, Mohammed A. Ismail, Dina A. Abdelgalil

**Affiliations:** Department of Chemistry, Faculty of Science, Mansoura University, Mansoura, 35516 Egypt

**Keywords:** Cellulose, Diaminoguanidine, Adsorption, Heavy metals, ICP-AES

## Abstract

In this study, the novel adsorbent diaminoguanidine-modified cellulose (DiGu.MC) was synthesized to extract mercury, copper, lead and cadmium ions from aqueous solutions and environmental water samples. The synthetic strategy involved oxidizing cellulose powder into dialdehyde cellulose (DAC) and reacting DAC with diaminoguanidine to create an imine linkage between the two reactants to form diaminoguanidine-modified cellulose (DiGu.MC). The structure and morphology of the adsorbent were studied using a variety of analytical techniques including Fourier transform infrared spectroscopy (FTIR), Scanning electron microscopy (SEM) and Brunauer–Emmett–Teller (BET) surface area measurements. Adsorption of mercury, copper, lead, and cadmium ions was optimized by examining the effects of pH, initial concentration, contact time, dose, temperature and competing ions. Under optimal adsorption conditions, the adsorption capacities of Cu^2+^, Hg^2+^, Pb^2+^, and Cd^2+^ were 66, 55, 70 and 41 mg g^−1^, respectively. The adsorption isotherm is in very good agreement with the Langmuir isotherm model, indicating that a monomolecular layer is formed on the surface of DiGu.MC. The kinetics of adsorption are in good agreement with the pseudo-second kinetics model that proposes the chemical adsorption of metal ions via the nitrogen functional groups of the adsorbent. Thermodynamic studies have confirmed that the adsorption of heavy metals by DiGu.MC is exothermic and spontaneous. Regeneration studies have shown that the adsorbent can be recycled multiple times by removing metal ions with 0.2 M nitric acid. The removal efficiency for regeneration was over 99%. DiGu.MC is introduced as a unique adsorbent in removing mercury, copper, lead and cadmium with a simple synthetic strategy, with cheap starting materials, a unique chemical structure and fast adsorption kinetics leading to excellent removal efficiency and excellent regeneration. The mechanism of adsorption of the investigated heavy metals, is probably based on the chelation between the metal ions and the N donors of DiCu.MC.

**Supplementary Information:**

The online version contains supplementary material available at 10.1186/s13065-022-00857-3.

## Introduction

Throughout the years, several techniques have been applied to extract heavy metals from polluted water including membrane filtration, adsorption, chemical precipitation, flotation, solvent extraction, chemical precipitation, coagulation, flocculation and ion exchange [1–14]. Adsorption techniques are believed to be the most efficient, the most cost effective, and the most ecofriendly [15–17]. Recently, biopolymers such as cellulose have proven to be excellent adsorbents of heavy metals [18, 19]. Nanocellulose and its derivatives have unique advantages such as low density, biodegradability, abundance, possibility of reuse and the ease to be functionalized and chemically modified [20–22]. Cellulose has sorption capabilities in both forms: natural and modified [22, 23]. Cellulose was used natively without modifications as an adsorbent for several materials such as metal ions, water vapor and dyes [22, 24–26].

On the other hand, modifications of cellulose can be carried out to achieve enhanced physical and chemical properties. For example, introduction of Polyethyleneimine to natural cellulose resulted in better adsorption of mercury [27], as well as of Cr^3+^ and Fe^3+^ [28] than unmodified cellulose. Also, cellulose modified with glycidyl methacrylate-and functionalized by imidazole has revealed significant removal of Ni^2+^ from aqueous solutions [29]. Complexation between cellulose and ethylene diaminetetraacetic dianhydride has as well enhanced the adsorption behavior of cellulose towards Cu (II), Cd (II), and Pb (II) ions [30]. Moreover, aminoguanyl modified cellulose represent a significant potential of metal ion extraction including Cu^2+^, Cd^2+^, Hg^2+^, Pb^2+^and Zn^2+^ [31]. In a commonly applied procedure, cellulose is selectively oxidized by periodate salts such as potassium periodate [32]. Potassium periodate has been chosen in particular for cellulose oxidation to guarantee the oxidation of two neighbouring carbon atoms into two aldehyde moieties as confirmed in previous work [33].

In this work, the novel adsorbent DiGu.MC was synthesized from cellulose after oxidation to incorporate diaminguanidine (DiGu) as a chelating ligand for the removal of heavy metals ions from natural water samples. The chelating ligand DiGu was chosen due to its abundant nitrogen functional groups, cost efficiency compared to other ligands, and its potential practical applications in the removal of heavy metals from wastewater. To the best of our knowledge, the modification of dialdehyde cellulose (DAC) with diaminoguanidine (DiGu) is scarcely reported in the literature. Moreover, no studies were published concerning the use of DiGu.MC in the removal of heavy metals from environmental samples. This work pursues an effective way to create surface modified cellulose powder with a multi-dentate chelating agent as a potential novel adsorbent for toxic metal ions.

In the present study, the adsorption characteristics of the modified cellulose (DiGu.MC) and its application to the preconcentration and separation of traces of four heavy metals viz., Cu(II), Hg(II), Pb(II) and Cd(II) from natural water samples prior to their ICP-AES determination have been described. Compositional and morphological aspects were investigated using various instrumental techniques.

## Experimental and methods

### Materials

Cellulose powder (microgranular, C6413), potassium metaperiodate (KIO_4_), *N*,*N*-Diaminoguanidine monohydrochloride, CuSO_4_, HgCl_2_, CdCl_2_ and Pb(NO_3_)_2,_ Triethylamine and ethanol were purchased from Sigma Aldrich and were used as received with no further modifications or purification procedures.

### Preparations

#### Preparation of dialdehyde cellulose (DAC)

The oxidized cellulose (DAC) is being prepared according to previously reported work of Kennawy et al. [24] In this process, native cellulose powder was subjected to oxidation by potassium periodate as follows: 0.5 g of cellulose powder was placed in 100 mL of distilled water and 1 g of potassium periodate was added to the solution, the mixture was then left to stir at temperature of 45 °C for 6 h. The formed dialdehyde cellulose (DAC) was collected by filtration and left to dry at room temperature.

#### Determination of aldehyde content

0.1 g dialdehyde cellulose (dry basis) was added to a 100 mL aluminum covered Erlenmeyer flask and 25 ml 0.25 M hydroxylamine hydrochloride-solution was added followed by stirring for 2.5 h in room temperature. This was performed in duplicate for the dialdehyde cellulose fibers obtained from each oxidation test. The fibers were then filtered off on an oven-dry and pre-weighed filter paper (7.0 cm, Munktell, Sweden), and the filter paper with the fibers was subsequently dried in 378 K for 1 h to determine the exact mass weight. The filtrate was titrated back to pH 4.0 with 0.1 M NaOH until the red-to-yellow end point was achieved. Since the color change was hard to read, a pH meter (Five Go pH, Mettler Toledo LE438, Switzerland) was used as well to verify the pH, in order to get an adequate result. A control test was also performed using native pulp. In this case a volume of 15 ml of the disintegrated 1% pulp solution was filtered off and the fibers used for further reaction with 0.25 M hydroxylamine hydrochloride. The control test was performed in duplicate as well.

The degree of oxidation, i.e. the aldehyde content, was calculated from the amount of sodium hydroxide required to reach a filtrate pH of 4.0 in accordance with Eq. 1:

1 $${\text{AC}}\% = ({\text{M}}_{{{\text{NaOH}}}} ({\text{V}}_{{{\text{sample}}}} {-}{\text{V}}_{{{\text{control}}}} ){16}0)/{\text{m}}) \times {1}00,$$

where M NaOH is 0.1 M, m is the dry weight of the DAC sample (g), and Mw is the molecular weight of the repeating unit, (C_6_H_8_O_10_) n, in DAC (160.124 g/mol). The consumption of NaOH is recorded as V_sample_ and V_control_ (L) [34, 35].

#### Preparation of diaminoguanidine modified cellulose (DiGu.MC)

0.75 g of diaminoguanidine was added to 0.5 g of DAC and immersed in 100 ml of ethanol in the presence of triethylamine. The reaction mixture was allowed to reflux at temperature of 80 °C for 6 h, following the procedure of dialdehyde-cellulose condensation reported in the mentioned studies [36, 37]. An orange yellow powder of the diaminoguanidine modified cellulose (DiGu.MC) was obtained and used in further adsorption procedure.

### Instrumentation

The percent composition of the native cellulose oxidized cellulose and DiGu.MC samples were determined on a Perkin–Elmer 240C Elemental Analytical Instrument (USA). To investigate the successive functionalization of the substrate cellulosic fibers until he DiGu-MC is obtained, an attenuated total reflectance (ATR) supported Perkin–Elmer Fourier-Transform Infrared (FT-IR) spectrometer (USA) was utilized. The surface morphology of the samples was determined using a scanning electron microscope (SEM) (FEI Quanta-200 FEI Company, The Netherlands). The fibers were sputtered and coated with gold before examinations. The concentrations of heavy metals in the solutions before and after removal experiments were quantified by Agilent's 5100ICP-OES (Agilent technologies. Melbourne, Australia). The TGA and DTA were recorded using Thermo analyzer Shimatzu DT40 (Japan) within a temperature range between 30 and 800 °C with 5 °C temperature break and under 20 mL/min flow rate of N_2_. The surface area of the modified cellulose was determined using Brunauer–Emmett–Teller (BET) analysis.

### Adsorption and desorption experiments [21]

Adsorption and desorption of heavy metals from contaminated water were determined in the experiments by using 5 mL of toxic metal solution and 5 mg of the adsorbent in 20 mL glass vials at various pH, initial concentrations, and contact time. The samples were acidified with 2% HNO_3_ before analysis, and ICP-OES was used to measure the concentration of heavy metal ions in the solutions after removal or desorption experiments at ppm levels. The adsorption capacity, removal efficiency, desorption capacity, and desorption efficiency were calculated from the following Eqs. (2)–(5), respectively.

2 $$\mathrm{qe} =\frac{(\mathrm{Ci}-\mathrm{Ce})\mathrm{V}}{m},$$

3 $$\mathrm{R\%}=\frac{(\mathrm{Ci}-\mathrm{Ce})}{\mathrm{Ci}}\times 100,$$

4 $$\mathrm{qd} =\frac{\mathrm{CdV}}{m},$$

5 $$\mathrm{De\%}=\frac{\mathrm{qd}}{\mathrm{qe}}\times 100,$$

where q_e_ is the adsorption capacity (mg/g), C_*i*_ is the initial concentration of heavy metal ions in the solution (mg/L), C_*e*_ is the equilibrium concentration (mg/L) after adsorption, V is the volume of the solution of metal ions (L) and m is the mass of the adsorbent (g), % R_e_ is the percentage of removal (%), qd is the desorption capacity in (mg/g), C_*d*_ is the concentration of heavy metal ions in the eluent in (mg/L) after the adsorption experiment, and % D_e_ is the desorption efficiency (%) [21].

### Batch experiments

Batch experiments were carried out to achieve the optimum conditions that lead to the highest adsorption records. An initial mass of 0.05 g of DiGu.MC (except for adsorbent dose effects study, mass was varied between 0.001 g to 0.05 g) is added to 50 ml of 50 mg.L^−1^ of metal ion solution, pH was extended from 1 to 6 by addition of 0.1 M NaOH and/or 0.1 M HCL.

Initially, the temperature was adjusted to 25 °C, and then differentiated to 35 °C, and 45 °C to study temperature effect. Initial metal ion concentration ranged between 50–250 mg.L^−1^. Contact time was first set to 6 h and then extended between 1–24 h to evaluate the contribution of contact time to adsorption behavior. Concentrations of Cu^2+^, Pb^2+^ and Cd^2+^ ions in each experiment were measured by ICP-AES.

Then, adsorption capacity (q_e)_ and the removal efficiency (R %) was estimated by Eqs. (2) and (3).

### Sample analysis

Tap water samples were collected (Mansoura University, Egypt). Surface natural water samples were collected from the Nile River (Damietta branch, Egypt) and seawater samples (Alexandria City, Egypt). All samples were filtered using a sintered glass G4 filter. All the selected samples were acidified with concentrated nitric acid to pH ~ 2 and then preserved in polyethylene vessels for further use. The organic matter was digested before the separation process; 0.5–1.0 g of K_2_S_2_O_8_ was added to one liter of the selected water sample and the mixture was heated for 30 min at 95 °C. After cooling to room temperature, 50 mg of DiGu.MC was added to a series of transparent stoppered bottles containing different concentrations of metal ions 0.0, 10 and 20 μgl^−1^at 25 °C and optimum pH conditions. The stoppered bottles were stirred at 150 rpm on an equilibrated shaker for 30 min; then filtered. To the filtrate another 30 mg of DiGu.MC was added and the pH was controlled again. The sample was stirred again for 15 min and filtered. Both residues were gathered and the collected metal ions were released by 10 ml of 2 M HNO_3_, to give a concentration factor of 100-fold.

## Results and discussion

### Materials’ design

#### Synthesis of dialdehyde cellulose (DAC)

**Fig. 1:**
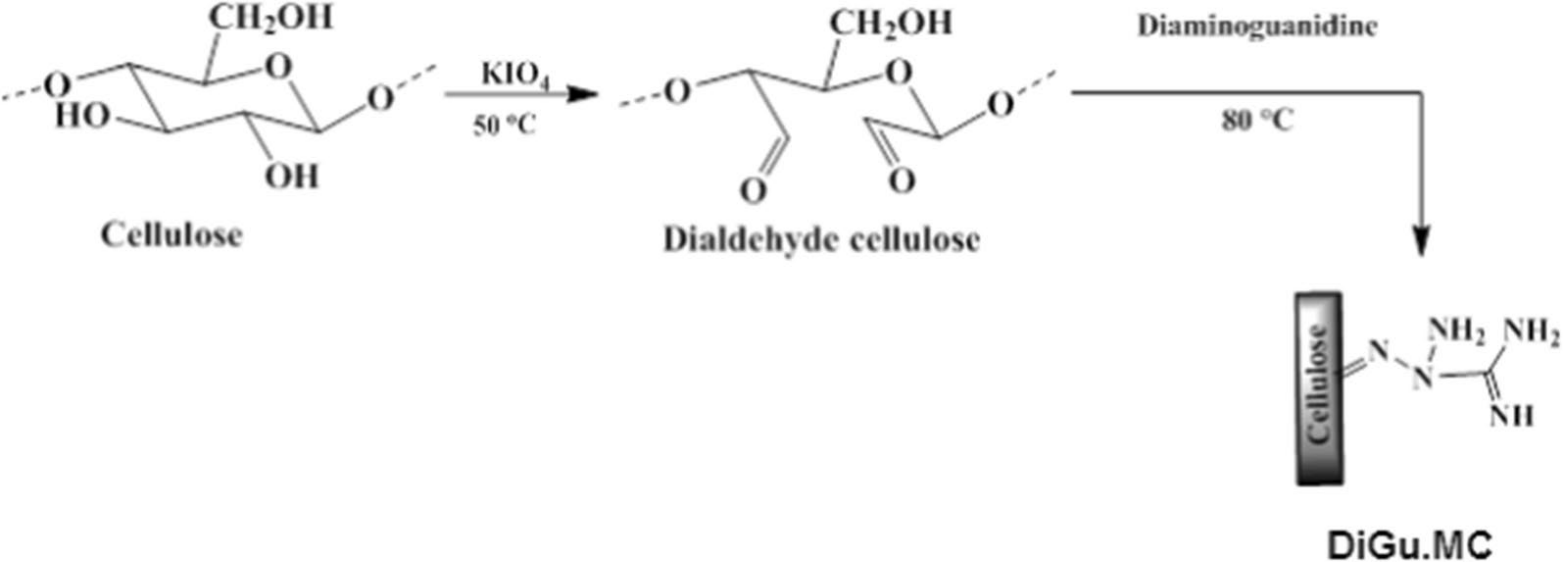
Synthesis of DiGu-MC

Dialdehyde cellulose is successfully prepared by potassium periodate oxidation which produced selective cleavage of the two secondary hydroxyl groups in C_2_–C_3_ vicinal hydroxyl groups in glucopyranose unite in cellulose chains; giving ring-opened product with dialdehyde groups as in Fig. 1. In this method large numbers of dialdehydes are introduced in cellulose chain with high selectivity and high yield. Aldehyde content (AC%) represents the oxidation degree (the percentage of monosaccharide units which reacted with periodate) [38]. The average AC% from the periodate oxidation of cellulose is 39.5%, Table 1.

**Table 1:** Volumetric titration of dialdehyde-cellulose (DAC) for determination of average aldehyde content percentage AC%

V_control_ (ml)	V_sample_ (ml)	C_NaOH_ (M)	m (gm)	AC %
0	2.4	0.1	0.1	38.4
0	2.5	0.1	0.1	40
0	2.5	0.1	0.1	40
Average AC%				39.5

#### Synthesis of DiGu.MC

Oxidation was followed by the reaction with diaminoguanidine at 80 °C for further chemical modification. The prepared DiGu.MC is solid powder orange -yellow in color. The solubility of DiGu.MC in water was tested by suspending a 1.00 g sample of DiGu.MC in 50.0 mL water. The suspension was stirred for approximately 3.0 h, and then the resulting solid was collected via filtration and dried. No reduction in the total mass was observed. Figure 1 represents the synthetic steps of DiGu-MC formation.

### Digital photographs

**Fig. 2:**
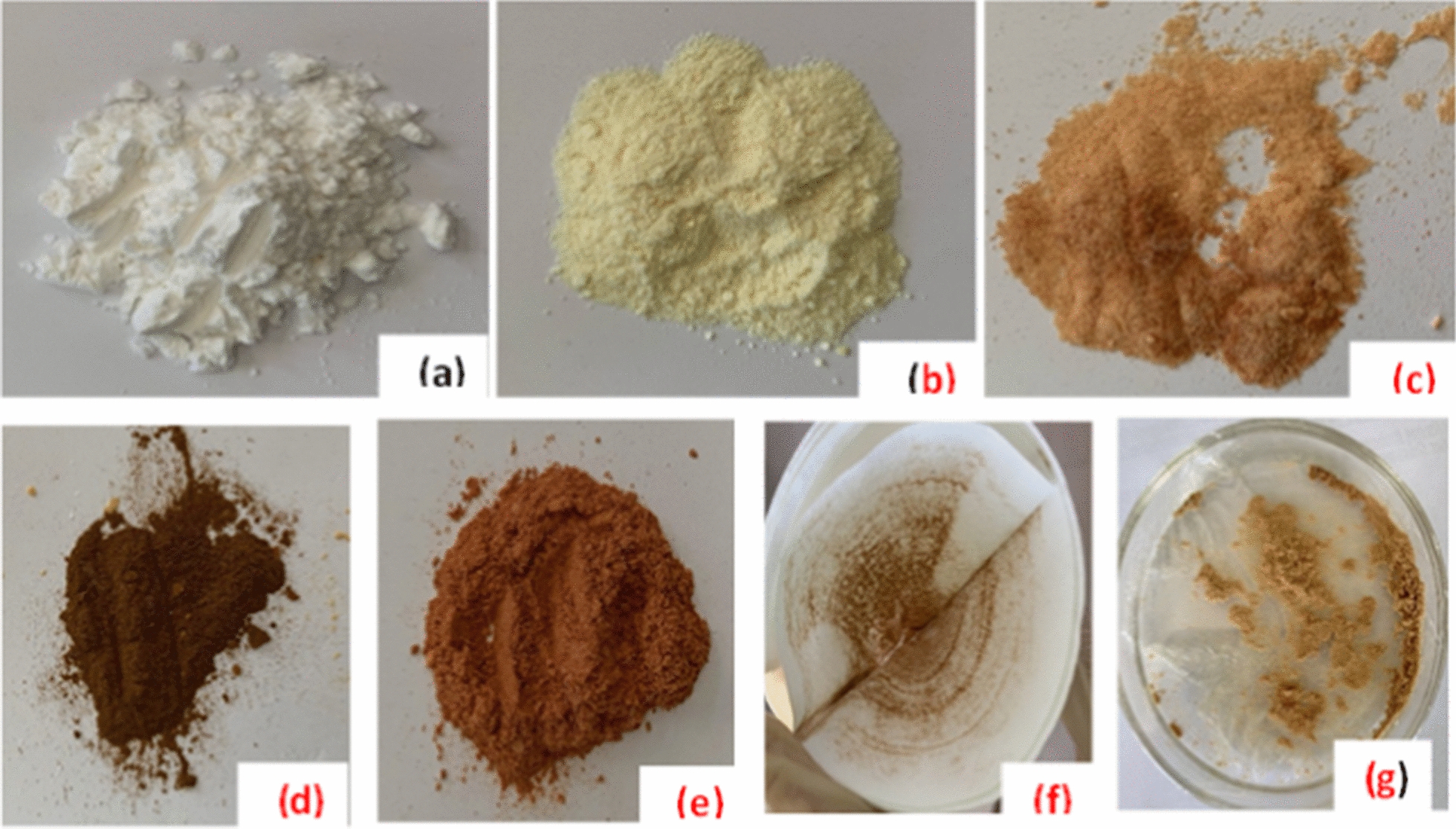
Digital photographs of: **a** Native cellulose powder, **b** oxidized cellulose, **c** modified cellulose (DiGu.MC), **d** Cu(II)-DiGu-MC, **e** Hg(II)-DiGu-MC, **f** Pb(II)-DiGu-MC and **g** Cd(II)-DiGu-MC

The digital photographs of native cellulose, oxidized (DAC), modified (DiGu.MC) and metal loaded DiGu.MC (Cu(II)-DiGu-MC, Hg(II)-DiGu-MC, Pb(II)-DiGu-MC and Cd(II)-DiGu-MC) are shown in Fig. 2a–g, respectively. The photographs showed obvious colour changes of the DiGu.MC before metal uptake (light brown, Fig. 2c) compared to DiGu.MC after metal uptake (Fig. 2d–g). These results indicated the tendency of the modified cellulose towards the adsorption of investigated metal ions [21].

### Characterization

#### Elemental analysis

The C, H and N elemental percent composition of the native cellulose along with the DiGu-MC are given in Table 2. As it can be clearly seen, unlike native cellulose DiGu.MC contain nitrogen (N). The existence of nitrogen (N) within DiGu-MC in a considerable percentage (24.76%) could be taken as clear evidence for the successful incorporation of the diaminoguanidine units within the chemical structure of the modified cellulosic fibers. The concentration of the inserted diaminoguanidine units was calculated to be approximately 2.4336 mmol g^−1^.

**Table 2:** Elemental composition of native and diaminoguanidine modified cellulose

	Carbon (%)	Hydrogen (%)	Nitrogen (%)
Native Cellulose	44.5	7.14	–
DiGu-MC	27.99	7.59	24.76

#### Scanning electron microscopy (SEM)

**Fig. 3:**
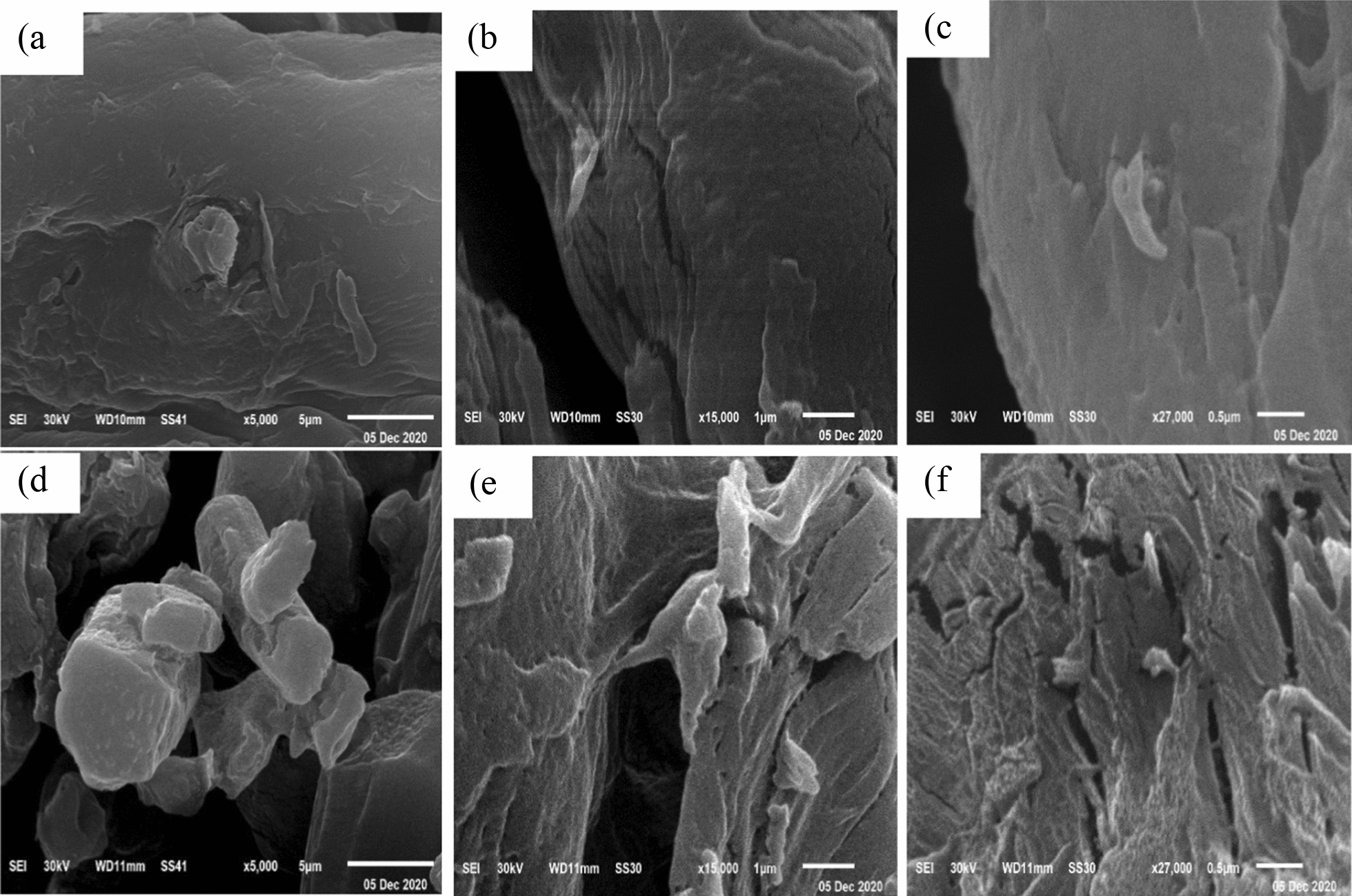
SEM images of DAC at: **a** 5000 ×, **b** 15000 ×, **c** 27000 ×; SEM images of DiGu.MC at **d** 5000 ×, **e** 15,000 × and **f** 27000 ×

Scanning electron microscope had been used to visualize the morphological appearances of each of DAC and DiGu-MC at magnifications of 5000X, 15000X and 27000X and the images of SEM are displayed in Fig. 3. The DiGu.MC fibers exhibited a rough surface with an irregular appearance compared to the DAC fibers. This could be attributed to the morphological changes that may take place within the fiber's structure during the chemical interaction with diaminoguanidine. This difference is also clarified by the color of each fiber. As it can be seen from Fig. 2; the DAC fibers (Fig. 2b) displayed a pale yellow color while the DiGu.MC fibers (Fig. 2c) were brown. This finding can imply the formation of a polymeric DiGu.MC.

#### Brunauer–Emmett–Teller (BET) analysis

The BET analysis has provided additional information about surface properties as listed in Additional file 1: Table S1. The results obtained showed that DiGu-MC demonstrated a surface area of 3.402 m^2^g^−1^ which is low as compared to the surface area of native cellulose. The decrease of the specific surface area after chemical modification may be due to the covering of cellulose pores by anchoring of diaminoguanidine moieties which reduced the adsorption of N_2_ molecules used in the surface area measurement process. The relatively low surface area of the functionalized fibers indicated that the adsorption process occurs mainly through the coordination of the funtional groups of diaminoguanidine with the metal ions.

#### FTIR spectra

**Fig. 4:**
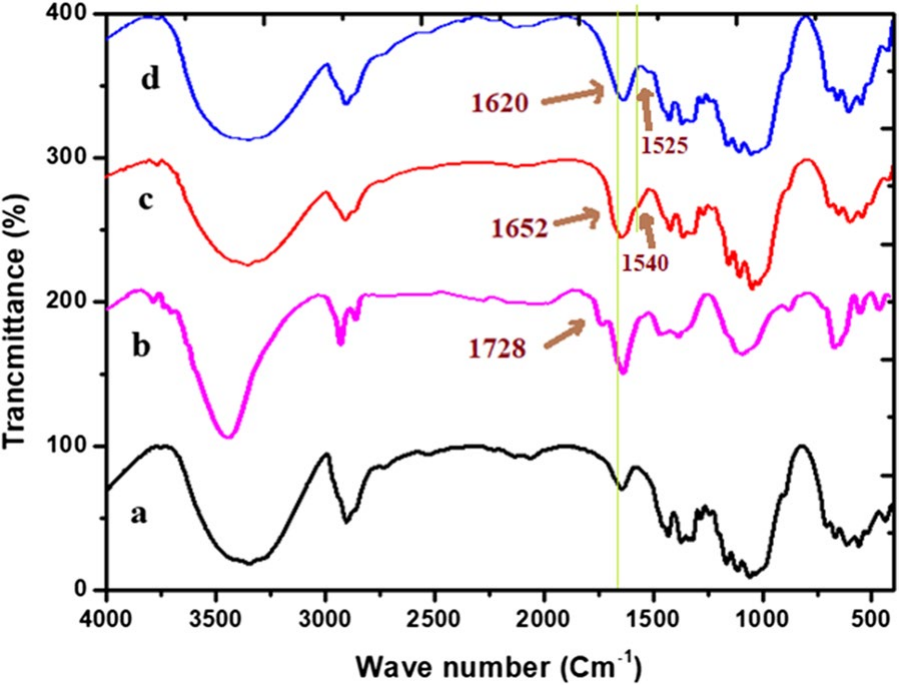
FTIR spectra of (**a**) native cellulose, (**b**) oxidized cellulose, (**c**) DiGu.MC and (**d**) Cu(II)-DiGu.MC

The FTIR of the successive steps that have been involved during the preparation of DiGu-MC are all represented in Additional file 1: Fig. S1a–d and collected in Fig. 4.

##### The FTIR of the native cellulose

The IR spectrum of the native unmodified cellulose (Additional file 1: Fig. S1a; Fig. 4a) represents some characteristic peaks in the range of 1000–1200 cm^−1^ that correspond to C–O stretching. While the peaks present at 1260–1410 cm^−1^ are attributed to O–H bending vibrations, and those present in the range of 3600–3200 cm^−1^ are corresponding to O–H stretching vibrations. Other peaks present at 2700–3000 cm^−1^ are due to C–H stretching [38].

- The FTIR spectrum of the DAC
  The FTIR spectrum of the DAC (Additional file 1: Fig. S1b; Fig. 4b) showed an extra moderately sharp peak at approximately 1728 cm^−1^ which is due to stretching vibration of carbonyl groups (C = O) formed upon oxidation [39].
- The FTIR of DiGu.MC (Additional file 1: Fig. S1c; Fig. 4c)
  After introduction of diaminoguanidine to the DAC, it was observed that the characteristic peak of the oxidized cellulose was weakened and broadened to cover the range of 1610 to 1670 cm^−1^ which could be attributed to the C=N group which is formed between the amino groups of diaminoguanidine and carbonyl groups of the oxidized cellulose [34] The strong peak at 3448 cm^–1^ and the peak at 1540 cm^–1^ are due to bending vibrations for N–H bond. The peaks at 3392 and 3300 cm^–1^ are due to the presence of the NH_2_ group.
  Therefore, FTIR results indicate the successful insertion of new functional groups to the natural cellulose upon oxidation and reacting with diaminoguanidine.
- FTIR of DiGu.MC-Cu(II) (Additional file 1: Fig. S1d, Fig. 4d)
  The IR spectrum of DiGu.MC adsorbent was compared with those of its complex with Cu(II) ions after complete dryness in a vacuum dessicator for 24 h, Fig. 4d. The main characteristic peaks of azomethine presented an obvious shift upon complexation with Cu^2+^ ions. Thus, the value of stretching vibrations of vibrations of C=N at ~ 1655 cm^−1^ was moved to lower value at 1620 cm^−1^. Additionally, the bands corresponding to -NH and -NH_2_ in the DiGu.MC adsorbent were shifted in the spectrum of DiGu.MC-Cu(II) complex.

The combination of the abovementioned characterization results, particularly FTIR spectra, and SEM provide strong evidence for the formation of DiGu.MC through two main steps. The first step is to convert cellulose into DAC, and the second step involves chemical reactions between −NH_2_ groups from the diaminoguanidine chelating ligand and the carbonyl group of DAC to form DiGu.MC.

### Thermogravimetric analysis (TGA)

The thermal stability of any adsorbent is an essential parameter that influences its efficiency. Thermal gravimetric analysis (TGA) was conducted to investigate the thermal decomposition of the modified cellulose (DiGu-MC) before and after being loaded with the adsorbed metal ions. As shown in Additional file 1: Fig. S2, over temperature range of (0–200) ^o^C there was very slight weight loss that actually starts after 100 ^o^C mainly due to the evaporation of water moieties. Weight loss starts to increase approximately from temperature 250 °C to about 450 °C due to pyrolysis of the samples. DiGu.MC before and after adsorption of metal ions represent very similar decomposition behavior and has confirmed to be thermally stable. Thermograms of natural cellulose reveal two thermal decomposition steps that usually yield levoglucosan and anhydrocellulose [34, 40]. Whereas, the obtained thermogram of DiGu.MC in Additional file 1: Fig. S2a shows four thermal decomposition stages which in turn confirms the occurrence of compositional modification of natural cellulose. At 995 °C the final remaining weight of DiGu.MC was 44.28% indicating the significant thermal stability of DiGu.MC at very high temperatures. After adsorption of metal ions, the final remaining weights of metal-DiGu-MC complexes at 995 °C were 48.6%, 33.7%, 53.3%, 0.016% for Cu-DiGu.MC, Hg-DiGu.MC, Pb-DiGu.MC and Cd-DiGu.MC, respectively. The decreased value of the remaining weight in case of mercury and cadmium compared to DiGu.MC suggests that Hg-DiGu.MC and Cd-DiGu.MC complexes are less thermally stable than DiGu.MC in addition to probable occurrence of catalytic degradation during their adsorption on DiGu.MC [34]. While Cu-DiGu.MC and Pb-DiGu.MC complexes show higher remaining weight compared to DiGu.MC at the same temperature which refers to their higher thermal stability than that of DiGu.MC.

### Solid phase extraction (SPE)

In the present work a batch mode adsorption process is used in which known concentrations of the metal ions under investigation (Cu(II), Hg(II) Pd(II) and Cd(II)) were dosed with a definite mass of DiGu.MC at the optimum parameters for separation. At equilibrium, filtration is carried out. Then, the filtrate was subjected to analysis ICP-AES to determine the residual amounts of the investigated metal ions. The various experimental variables affecting the process of adsorption of the investigated metal ions using the DiGu.MC adsorbent, were thoroughly investigated to determine the optimal extraction conditions that lead to the highest adsorption efficiency viz., the solution pH, the adsorbent dosage, the contact time, the temperature, evaluated to.

#### Point of zero charge (PZC)

The point of zero charge (PZC) is an important parameter for the characterization of the sorbate as well as illustrating the affinity of the sorbate to sorbent's surface. The PZC of DiGu.MC was investigated according to the reported study [34]. The value of pH was changed from 1 to 7; and the PZC for DiGu.MC was determined. The value of PZC for the DiGu.MC is found to be located at pH 5.80. According to this result the surface of the Di.Gu.MC displayed positive charges at a pH below 5.8, while at a pH above 5.8 the surface of Di.Gu.MC displayed negative charges.

#### Effect of pH

**Fig. 5:**
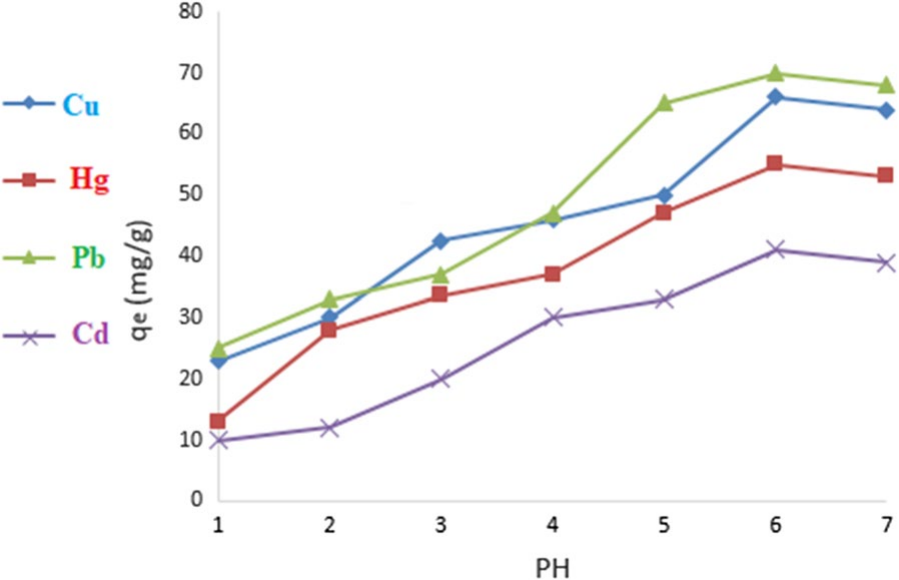
Effect of pH on adsorption of heavy metals by DiGu-MC

The effect of pH on the removal of Cu(II), Hg(II), Pd(II) and Cd(II) metal ions on DiGu.MC was studied using a pH range of 1−7. The results plotted in Fig. 5 indicate that as the pH of the solutions increases from 3 to 6, the adsorption capacity increased to the maximum values at pH 6. The low adsorption capacities for the investigated metal ions at low pH were attributed to the presence of hydrogen ions that would interfere with the adsorption process via competition with the metal ions for the sorbent surface binding sites. Above pH value of 6.0, the rate of removal of metal ions started to decrease which could be attributed to the formation of metal oxides and hydroxides.

#### Effect of adsorbent dose

**Fig. 6:**
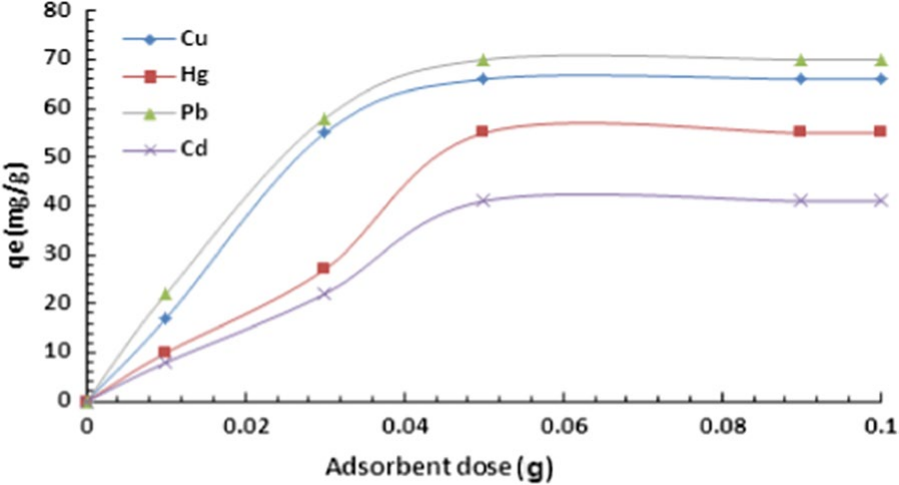
Effect of adsorbent dose on adsorption of heavy metals by DiGu-MC

The dose of adsorbent was varied from 0.01 g to 0.1 g and the adsorption capacity was estimated for each dose. Figure 6 describes the relationship between adsorbent dose and adsorption capacity of DiGu.MC. It can be concluded that adsorption capacity increases as the adsorbent dose increases in a direct relationship this can be simply due to the increased number of active sites present. Then, when saturation by heavy metals is achieved the adsorption capacity turns to remains constant as the adsorbent dose increases. DiGu.MC has shown saturation values at dose of 0.05 g for all the adsorbed metals.

The results could be attributed to the adsorption's mechanism, which is goverened by diffusion and surface coordination. As the amount of added sorbent increases, the number of available binding sites increases; thus, the removal percentage of metal ions increases. With full saturation of the coordination sites, another adsorption process becomes the dominant factor, i.e., the diffusion process, which is primarily controlled by the concentration of the metal ions on the polymer surface and the free metal ions; when these two concentrations become equal, adsorption process comes to an end. The results summarized in Fig. 6 showed that the optimum adsorbent dosage was approximately 50.0 mg. The obtained results proved that DiGu-MC is a highly efficient adsorbent for heavy metals' removal, even at very low dosages.

#### Effect of contact time and adsorption kinetics

**Fig. 7:**
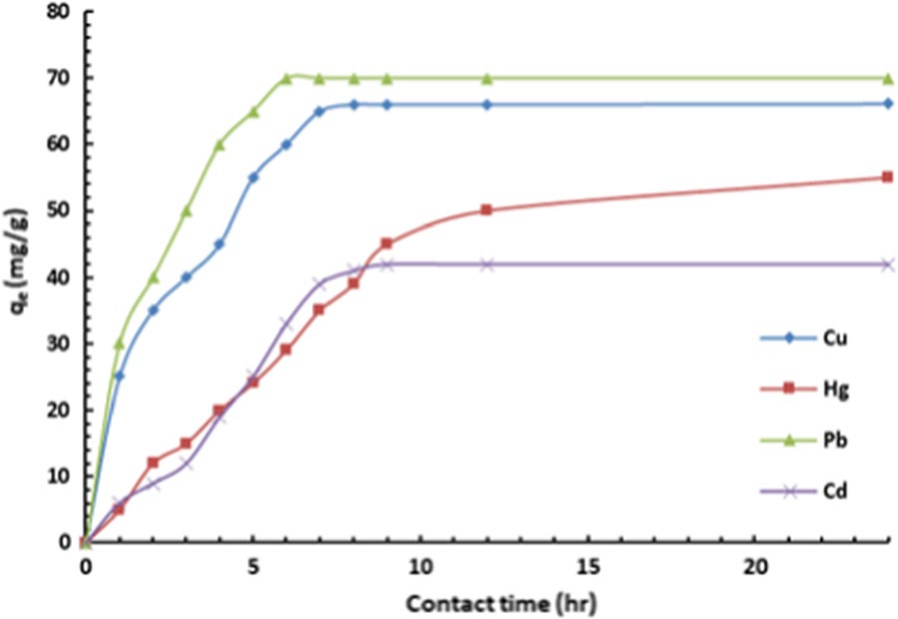
Effect of contact time on adsorption of heavy metals by DiGu-MC

The adsorption kinetics' uptake of Cu(II), Hg(II), Pb(II) and Cd(II) by the modified DiGu.MC were investigated by monitoring the adsorbed metal ions' amounts within predetermined time intervals from 1 to 8 h and the results are presented in Fig. 7. As it can be seen, the uptake of the metal ions demonstrated an initial rapid profile for Cu(II) and Pb(II) ions and a slightly slower initial profile for Hg(II) and Cd(II); and the equilibrium was reached within around 6 h for all metal ions.

To clarify the rate and kinetics of adsorption process, several adsorption kinetic models such as pseudo-first-order and pseudo-second-order models were used. The obtained kinetic data were used to explain the nature and mechanism of the uptake of the metal ions by DiGu.MC using both pseudo 1st order model and pseudo 2nd order mathematical equations Eqs. (6) and (7), respectively [36].

Pseudo-first-order linear equation:

6 $${1}/{\text{q}}_{{{\text{t}}({\text{ads}})}} = {\text{ k}}_{{1}} /{\text{q}}_{{{\text{e}}({\text{ads}})}} {\text{t }} + { 1}/{\text{q}}_{{{\text{e}}({\text{ads}})}} .$$

Pseudo-second-order linear equation:

7 $${\rm t}/{\rm q}_{\rm t}({\rm ads})=1/{\rm k}_{2}{\rm q}_{\rm e}^{2}({\rm ads})+ (1/{\rm q}_{\rm e}({\rm ads})){\rm t},$$

where q_e(ads)_ is the adsorption capacity at equilibrium, q_t(ads)_ is the adsorption capacity at time t, K_1_ is the adsorption rate constant of pseudo-first-order model and K_2_ is the adsorption rate constant of pseudo-second-order model.

**Fig. 8:**
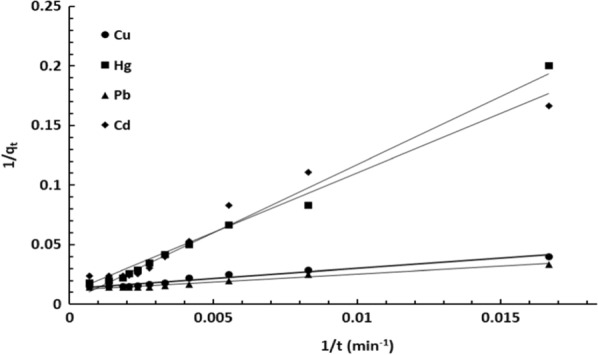
Pseudo-first order model

**Fig. 9:**
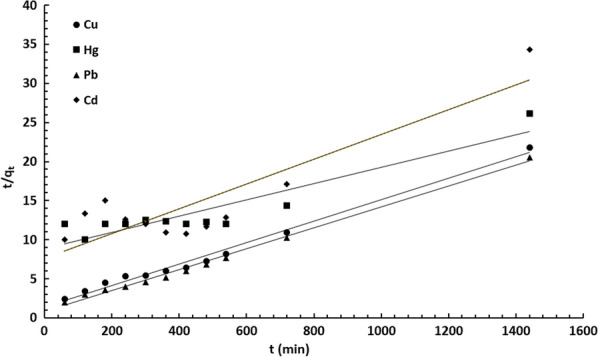
Pseudo-second order model

The kinetic parameters estimated from the two models are summarized in Table 3 and the plotted curves are shown in Figs. 8 and 9. Analysis of the obtained constants and parameters of the kinetic models clearly revealed that the pseudo-second-order model fits quite well with the experimental data (correlation coefficient R^2^ > 0.999). As shown in Table 3, the calculated maximum adsorption capacities of Cu(II), Hg(II), Pb(II) and Cd(II) were in good agreement with the experimental results. Therefore, the adsorption process occurs through the sharing or exchange of electrons between DiGu.MC and metal ions, resulting in the removal of the ions from water.

**Table 3:** Kinetic parameters for the adsorption of Cu(II), Hg(II), Pb(II) and Cd(II) by DiGu-MC

Adsorbate	Cu^2+^	Hg^2+^	Pb^2+^	Cd^2+^
Pseudo 1st order				
q_e(ads)_ (mg/g)	76.92	400	84.034	97.087
K_1_ (min^−1^)	130.938	4581.6	111.176	971.75
R^2^	0.9633	0.9886	0.9689	0.9549
Pseudo 2nd order				
Adsorbates	Cu^2+^	Hg^2+^	Pb^2+^	Cd^2+^
q_e(ads)_ (mg/g)	72.46	95.2	74.63	63.29
k_2_ (g/mg.min)	1.41 × 10^–4^	1.25 × 10^–5^	2.297 × 10^–4^	3.284 × 10^–5^
R^2^	0.9892	0.8517	0.994	0.7893

It can be noticed that adsorption of Cu^2+^, Hg^2+^, Pb^2+^ and Cd^2+^ by DiGu-MC fits well the pseudo-second-order kinetic model as the correlation coefficients (R^2^) were high approaching 1.0 and the theoretical equilibrium adsorption capacities q_e(ads)_ for each metal were consistent with the experimental records. Regarding rate constants (K_1_ and K_2_) calculated in each model, it can be clearly observed that rate constants calculated from first order model were high which means a slow adsorption rate which in turn doesn’t agree with experimental results. On the other hand, the rate constants derived from the second order model were much smaller which makes more sense and correlate with experimental results. It can be also concluded that chemisorption is the main dominant process and it is suggested to be as well the limiting step.

#### Effect of initial concentrations and adsorption isotherms

The effect of initial concentration of metals on adsorption was studied by varying initial concentration over the range of (50–250) ppm while all the remaining parameters were held constant. Additional file 1:Fig. S3 describes the relationship between initial concentration of metal ions and adsorption capacity of DiGu.MC. As shown in Additional file 1: Fig.S3, adsorption capacity increases as the initial concentration of metal ions increases till all the active sites of the adsorbent dose are occupied by metal ions then equilibrium is reached and the adsorption capacity remains constant as the initial concentration increases**.** The mathematical analysis of the isotherms was carried out by the well-known Langmuir and Freundlich models that are presented by Eqs. (8) and (9), respectively.

Linear equation of Langmuir isotherm model:

8 $${\text{C}}_{{\text{e}}} /{\text{q}}_{{\text{e}}} = \, \left( {{1}/{\text{K}}_{{\text{L}}} {\text{q}}_{{\text{m}}} } \right) \, + \, \left( {{\text{C}}_{{\text{e}}} /{\text{q}}_{{\text{m}}} } \right) .$$

Linear equation of Freundlich isotherm model:

9 $${\text{ln q}}_{{\text{e}}} = {\text{ ln K}}_{{\text{F}}} + { 1}/{\text{n ln C}}_{{\text{e}}} ,$$

where q_e_ is the adsorption capacity at equilibrium, K_F_ is Freundlich constant, n is the heterogeneity factor that reflects the energy distribution in bonds and C_e_ is metal concentration at equilibrium.

Where C_e_ is metal concentration at equilibrium, q_e_ is the adsorption capacity at equilibrium, K_L_ is the Langmuir constant and q_m_ is the maximum adsorption capacity of a single layer.

The values of the parameters resulting from the above equations are collected in Table 4. It can be observed that the results are consistent with the Langmuir model more than the Freundlich model with the observed higher R^2^ values suggesting the uptake of the metal ions by DiGu.MC.

**Table 4:** Langmuir and Freundlich constants for metal adsorption by DiGu-MC

Langmuir isotherm model	K_L_(L/g)	q_m_(mg/g)	R^2^	R_L_
Adsorbates				
Cu^2+^	0.323	101.01	0.9968	0.0122–0.0583
Hg^2+^	0.371	73.50	0.9965	0.0107–0.0512
Pb^2+^	0.497	112.36	0.9981	0.008–0.0387
Cd^2+^	0.067	64.52	0.9904	0.0563–0.2299

Freundlich isotherm model	K_F_	n	R^2^
Adsorbates			
Cu^2+^	81.48	35.84	0.8461
Hg^2+^	63.94	48.78	0.8606
Pb^2+^	91.49	33.33	0.8443
Cd^2+^	27.26	6.73	0.8167

The convenience of the adsorption process was then investigated by calculating (R_L_) which is the separation factor constant using Eq. (10).

10 $${\text{R}}_{{\text{L}}} = { 1}/ \, \left( {{1} + {\text{C}}_{{\text{o}}} {\text{K}}_{{\text{L}}} } \right),$$

where K_L_ is the Langmuir adsorption constant and C_o_ is the initial concentration of metal ions.

R_L_ values greater than 1.0 indicate unsuitability of the adsorbent, while R_L_ values between 0 and 1 indicate suitability of the studied adsorbent.

The derived parameters are listed in Table 4. From the listed estimated values, it was found that correlation coefficients were very high in Langmuir model which means that the experimental data significantly fit to Langmuir model. While R^2^ coefficients derived from Freundlich isotherm plot were much lower. In addition, the maximum adsorption capacity for a single layer (q_m_) obtained from Langmuir plot were all in agreement with experimental records which well confirms that adsorption process would be best described by Langmuir isotherm model. In addition, all the calculated values of R_L_ are significantly less than 1 confirming the suitability of DiGu-MC as an adsorbent for the studied metal ions.

#### Effect of temperature and adsorption thermodynamics

A thermodynamic study was conducted to check the spontaneity of the adsorption process.

In order to study the effect of temperature on adsorption of the studied metal ions, some thermodynamic properties were investigated including Gibbs free energy change (ΔG^o^), thermodynamic equilibrium constant (K_c_), standard entropy change (ΔS^o^) and standard enthalpy change (ΔH^◦^). The values of the mentioned thermodynamic parameters are calculated after plotting 1/T against LnK_c_ using Eqs. (11, 12 and 13).

11 $${\text{K}}_{{\text{C}}} = {\text{C}}_{{{\text{ad}}}} /{\text{ C}}_{{\text{e}}} .$$

12 $${\text{ln K}}_{{\text{C}}} = \Delta {\text{S}}^{{\text{o}}}_{{{\text{ads}}}} /{\text{ R}} - \Delta {\text{H}}^{{\text{o}}}_{{{\text{ads}} }} /{\text{RT}}{.}$$

R is gas constant (8.314 J/mol K).

13 $$\Delta {\text{G}}_{{{\text{ads}}}}^{ \circ } = \, - {\text{RT ln K}}_{{\text{C}}} ,$$

**Fig. 10:**
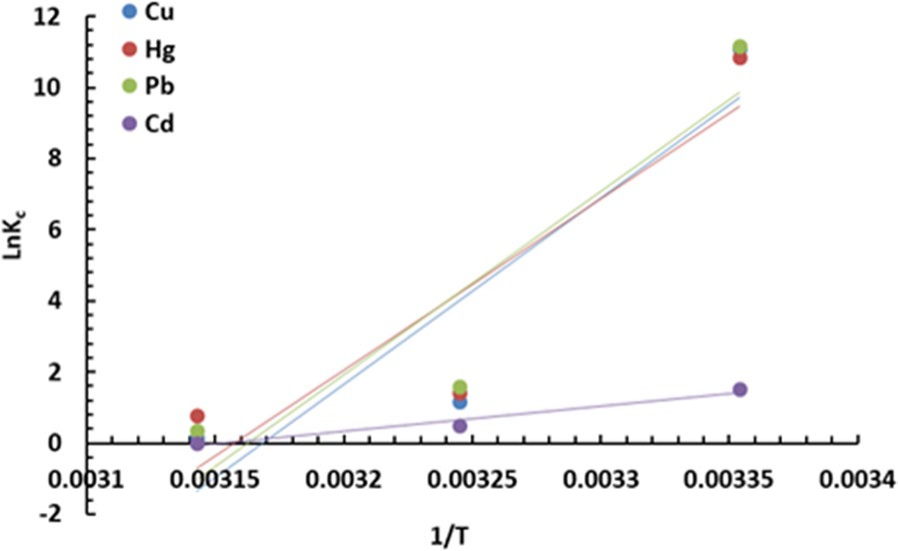
Relationship between LnKc and 1/T for adsorption of Cu^+2^, Hg^+2^, Pb^+2^ and Cd^+2^ by DiGu-MC

Figure 10 shows the plotted curves and the obtained values are all listed in Table 5. The investigated temperature range was 298 K to 318 K.

**Table 5:** Thermodynamic parameters of adsorption of Cu^+2^, Hg^+2^, Pb^+2^ and Cd^+2^ by DiGu.MC

	Ä${\text{G}}_{{{\text{ads}}}}^{ \circ }$ (KJ/mol)			Ä${\text{H}}_{{{\text{ads}}}}^{ \circ }$ (KJ/mol)	Ä${\text{S}}_{{{\text{ads}}}}^{ \circ }$ (J/mol K)
	298 K	308 K	318 K		
Cu^2+^	− 27.51	− 2.92	− 0.321	− 436.2	**− 1382.3**
Hg^2+^	− 26.82	− 3.55	− 1.99	− 400.3	**− 1264.1**
Pb^2+^	− 27.65	− 4.04	− 0.91	− 429.45	**− 1358.34**
Cd^2+^	− 3.76	− 1.25	0	− 59.96	− 189.135

Values of Gibbs free energy Δ${\text{G}}_{{{\text{ads}}}}^{ \circ }$ for all the adsorption process were negative indicating the spontaneity of the adsorption process under the investigated temperature range. Values of enthalpy change (ΔH^o^) were also negative for all the adsorption processes indicating the exothermic behavior of them. Standard entropy change (ΔS^◦^) is a measurement of randomness or energy distribution in the system. ΔS^◦^ values were all negative as well indicating the low randomness which reflects the strong affinity between DiGu.MC and the adsorbed metal ions, which is a very good indication of the adsorption efficiency of the adsorbent.

Thus, it can be concluded that high temperatures are not favorable for adsorption of the studied metal ions by DiGu.MC. So, adsorption experiments are more preferred to be conducted at moderately low temperatures such as 298 K which is the normal room temperature.

#### Effect of selected interfering ions:

Effect of the presence of foreign ions on adsorption of heavy metals was investigated under the optimum adsorption conditions. The removal percentage for each metal ion was estimated in the presence of 50 ppm of some interfering ions. Concentration of the interfering ions was exactly equal to the metal ion concentration. The results are all summarized in Table 6. It can be concluded from the obtained results that the presence of 50 ppm of cations such as Mg^2+^, Fe^2+^, Ca^2+^ and Al^3+^ and anions such as PO_4_^3−^, acetate, oxalate and edetate doesn’t significantly affect the removal of heavy metals by the prepared DiGu-MC adsorbent. The results give a promising indication about the selectivity of DiGu-MC which reveals its potential to work efficiently in more complicated media.

**Table 6:** Influence of interfering cations and anions on recovery percentage, *R* (%), of 10 μg ml^–1^ of each heavy metal ions using DiGu.MC. conditions: 0.4 g l^–1^ DiGu.MC, stirring time 60 min, pH 6 at 25 °C

Interfering ions	Concen (μg ml^–1^)	% Removal			
		Cu^2+^	Hg^2+^	Pb^2+^	Cd^2+^
Mg^2+^	50	99.4	99.1	99.4	77.6
Fe^2+^	50	98.1	98.2	97.4	75.6
Ca^2+^	50	100	100	99.7	77.6
Al^3+^	50	97.7	94.2	98.2	74.7
${\text{PO}}_{4}^{3 - }$	50	98.6	98.4	99.1	76.5
Acetate	50	97.1	98.4	96.2	76.1
Oxalate	50	98.4	98.2	98.4	73.2
Edetate	50	96.2	97.1	96.1	72.6

### Proposed mechanism of adsorption

The adsorption process usually supports the mixed mechanisms of physisorption and chemisorption mechanism. The physisorption mechanism always considered the adsorption process done through electrostatic transactions between positive charges of metal ions and negative surface of adsorbents which are usually full of donating electrons groups such as hydroxyl group, amine group, carbonyl group…. etc. On the other hand, the chemisorption mechanism is explained as ion exchange or chelation interaction one. In the chelation mechanism, a covalent coordination bond can be generated between Lewis acids (heavy metals, which have vacant d-orbitals) and Lewis bases (atoms with a lone pair of electrons). Therefore, because DiGu.MC has active sites such as N, it is possible to act as Lewis bases and form stable coordination complexes with metal ions in the solution, which acts as Lewis acids.

**Fig. 11:**
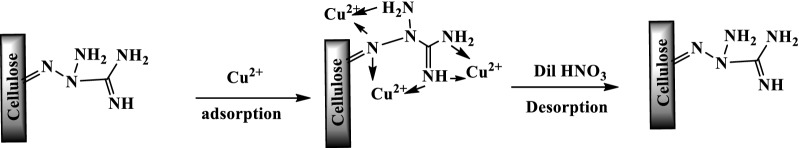
Proposed mechanism of adsorption and desorption of Cu^2+^ions with DiGu.MC

In this work, the mechanism of adsorption of the divalent metal ions onto DiGu.MC is proposed to be chemisorption mechanism. This proposal is based on the following findings:

1. DiGu.MC has active sites such as N; it is possible to act as Lewis bases and form stable coordination complexes with metal ions in the solution, which acts as Lewis acids.
2. The data obtained from kinetic section clarify that Di.Gu.MC adsorbent obeyed pseudo second order model which improved the chemisorption pathway.
3. These data agree with results of isotherm studies which indicated complete following of Di.Gu.MC adsorbents to the mono-layer Langmuir model against other model; the arrangement of adsorbates as only one layer completely support the chemisorption interaction suggestion.
4. In view of the IR results, it can be concluded that the coordination between DiGu.MC and the investigated metal ions would take place via the C=N, –NH and –NH_2_ groups of the DiGu.MC adsorbent. In this regard, the coordination number can be completed via the hydroxide ions and water molecules available at pH 6.0. The utilization of more than one DiGu.MC substrate per metal ion is unlikely because a steric effect arose from bonding to the surface of the cellulose base. By the same way, the appearance of new functional groups, at FTIR chart, were formed from the chemical treatment for DiGu.MC push in the way of chelating interaction between metal ions and Di.Gu.MC adsorbent. The possible structure to chelate copper (as representative example) is depicted in Fig. 11. The high affinity could be attributed to the high number of binding sites in DiGu.MC (as shown in Fig. 11).

### Selectivity studies of multi-metal solutions

A mixture of the four metals under study was prepared to investigate the adsorption efficiency of DiGu-MC in multi-component metal solutions. As shown in Table 7, removal percentages show the same trend of mono-component metal solutions with no obvious differences. The results also correlate with the conclusion obtained in interfering ion section that DiGu.MC would act efficiently in complicated multi-component samples. ICP–AES was used to determine the concentrations of the multiple metal ions in studied solutions.

**Table 7:** Removal of heavy metals from mixed metals solution by DiGu-MC

Sample	Metal ion	Initial concentration, µg l^-1 ^	Final concentration, µgl^-1^	Removal, %
Multi-component solution of (Cu2+, Hg2+, Pb2+ and Cd2+)	Cu^2+^	50.0	2.1	95.8
	Hg^2+^	50.0	0.6	98.8
	Pb^2+^	50.0	0.315	99.37
	Cd^2+^	50.0	14.5	71.00

### Desorption and reusability of DiGu-MC

To test the reusability of DiGu.MC, five cycles of adsorption–desorption have been carried out under the optimum conditions, using 5 ml of 0.2 M HNO_3_, the obtained results are shown in Table 8. From the results, it was clear that the adsorption efficiency of DiGu-MC was only slightly decreased after cycle five, the adsorbent maintained about 95% of its initial efficiency. These results prove that DiGu.MC adsorbent has tremendous reusability for Cu (II), Hg(II), Pb(II) and Cd(II) from aqueous solutions.

**Table 8:** Repeated adsorption cycles of Cu^2+^, Hg^2+^, Pb^2+^ and Cd^2+^ by DiGu-MC

Cycle number	Recovery (%)			
	Cu^2+^	Hg^2+^	Pb^2+^	Cd^2+^
**1**	99.6	99.1	99.2	98.1
**2**	98.7	98.3	98.4	97.3
**3**	98.4	97.7	96.7	96.4
**4**	97.8	96.1	95.8	95.6
**5**	96.6	95.1	95.0	94.7

### Applications in natural environmental water samples

The optimized experimental conditions were applied to real samples to evaluate the efficiency of the combined SPE-ICP-AES method for the pre-concentration and determination of trace Cu(II), Hg(II), Pb(II) and Cd(II) ions. The calibration curves were prepared with the standard solutions. The standard solutions (1.0 L) were treated under the experimental conditions optimized above. The analytical samples were tap water in our lab in Mansoura University, Nile water in Mansoura City and Mediterranean Sea water in Marsa Matrouh City. The analytical results are as shown in Table 9 From Table 9, it can be noticed that the concentrations of investigated Cu(II), Hg(II) Pd(II) and Cd(II) metal ions lie in the permissible accepted levels and are in agreement with those reported previously.

**Table 9:** Multi-elements analysis of natural water samples using ICP-AES for the determination of heavy metal ions in μgl^−1^ (ppb), after preconcentration using DiGu.MC adsorbent

Sample	Metal ions	Spiked	Measured	Recovered	Recovery, %
Tap water	Cu(II)	0.00	2.3 (3.5–5.0)	00	00
		10	11.8	9.5	95.00
		20	21.9	19.6	98.00
	Hg(II)	0.00	ND	ND	ND
		10	9.60	9.60	96.00
		20	19.80	19.80	99.00
	Pb(II)	0.00	4.4 (0.5–5.0)	00	00
		10	14.1	9.70	97.00
		20	24.00	19.60	98.00
	Cd(II)	0.00	0.30 (0.2–0.6)	00	00
		10	9.70	9.70	97.00
		20	19.80	19.5	97.50
Nile water	Cu(II)	0.00	3.60 (5.0–20.0)	00	00
		10	13.20	9.60	96.00
		20	23.00	19.40	97.00
	Hg(II)	0.00	0.1 (0.08–0.20)	00	00
		10	9.90	9.90	99.00
		20	19.30	19.20	96.10
	Pb(II)	0.00	2.50 (9.0–18.0)	00	00
		10	11.90	9.40	94.00
		20	22.10	19.60	98.00
	Cd(II)	0.00	0.13 (0.20–0.60)	00	00
		10	9.93	9.80	98.00
		20	19.63	19.50	97.5
Sea water	Cu(II)	0.00	31.70	00	00
		10	41.60	9.90	
		20	51.40	19.70	98.50
	Hg(II)	0.00	0.09	00	00
		10	9.94	9.85	98.50
		20	19.49	19.40	97.00
	Pb(II)	0.00	10.5 (1–28)	00	00
		10	20.00	9.50	95.00
		20	30.10	19.60	98.00
	Cd(II)	0.00	0.13 (0.2–1.0)	00	00
		10	9.93	9.80	98.00
		20	19.53	19.40	97.00

Conditions: pH = 6, 400 mg l^–1^ adsorbent, stirring time 60 min at 25 °C, *n* = 5. ND = not detected. All measured RSD ranges from 1 to 5%. The values in parentheses are the convenient or permissible values or values measured from previous work on the investigated elements

The recoveries were examined in the samples in which defined amounts of metal ions were spiked. The recoveries obtained were in the range of 95.00- 99.00%. The higher values of recovery (*R*, %) could be attributed to the strong chelation of the metal ions with the active N sites of the DiGu.MC adsorbent. This is also in agreement with the high charge densities of the studied metal ions. Accordingly, these elements can be preconcentrated and separated quantitatively by a single-step batch mode process.Additionally, the obtained results indicate that the proposed SPE-ICP-AES methodology could be successfully applied to the determination of trace amounts of Cu(II), Hg(II), Pb(II) and Cd(II) ions in real water samples.

### Comparison between adsorption capacity of DiGu-MC and other adsorbents

Number of published adsorbents and their adsorption capacities towards metal ions are compared to DiGu-MC as shown in Table 10. Comparing the adsorption capacities obtained from most of the mentioned studies, DiGu-MC was found to have higher adsorption capacity. Taking Cu^+2^ as an example, DiGu-MC has an adsorption capacity of 66 mg g^−1^ while all the listed methods revealed adsorption capacities ranged from 1.75 to 58.27 mg g^−1^.

**Table 10:** Comparison of the adsorption capacities of Cu^+2^, Hg^+2^, Pb^+2^ and Cd^+2^ by DiGu-MC with other published adsorbents

Metal ion	Adsorbent	Adsorption capacity q_e_ (mg g^−1^)	References
Cu^+2^	DiGu-Modified cellulose	66	Present work
	Cellulose modified with acrylic acid	17.2	[41]
	Microfbrillated cellulose modified with aminopropyltriethoxysilane	3.150	[42]
	Citric acid modified cellulose	24	[43]
	Pristine nanocellulose	20	[44]
	Cortex banana waste	36.0	[45]
	Cationic wheat straw	33.5	[46]
	Orange peels modified with HNo_3_ (0.1 M)	15.27	[47]
	Activated carbons using hazelnut shell	58.27	[48]
	Tobacco dust as a lignocellulosic source	36.0	[49]
	Peanut shells,	25.39	[50]
	Rice husk	30.0	[51]
	Oil palm shell	1.75	[52]
	Pomegranate peel	30.12	[53]
Hg^+2^	DiGu-Modified cellulose	55	Present work
	Guanyl modified cellulose	48.0	[34]
	Bamboo leaf powder as a cellulose source	27.11	[54]
	Bacillus subtilis biomass	68.5	[55]
	Eucalyptus bark	34.60	[56]
	Allium sativum L. extract	0.6497	[57]
	Silica gel modified with 2-(2-oxoethyl)hydrazine carbothioamide	37.5 3	[58]
	Magnetic nanoparticles doped with 1,5-diphenylcarbazide	44	[59]
Pb^+2^	DiGu-Modified cellulose	70	Present work
	S. bengalense extract modified with urea	12.65	[60]
	Sorghum bicolor L. modified with thiourea	17.82	[61]
	Pine cone powder modified with NaOH (0.01 M)	24.75	[62]
	Oil palm shell	3.39	[52]
	Cauliflower waste	47.63	[63]
	Acrylic acid modified cellulose	55.9	[41]
	Thiol- functionalized cotton as cellulosic biomass	10.78	[26]
	Tobacco dust as a lignocellulosic source	39.6	[49]
	Guanyl modified cellulose	52	[34]
	Citric acid modified cellulose	83	[43]
	Nano-TiO_2_	7.41	[64]
Cd^+2^	DiGu-Modified cellulose	41	Present work
	Tobacco dust as a lignocellulosic residue	29.6	[49]
	Guanyl modified cellulose	68	[34]
	T. aestivum Urea	9.22	[65]
	Rice husk modified with NaOH	20.24	[66]
	Rice husk modified with NaHCO_3_	16.18	[66]
	Orange peels modified with HNO_3_ (0.1 M)	13.7	[47]
	Cellulose derived from corn stalk	21.37	[67]
	Cellulose powder modified with acrylic acid	30.3	[41]
	Cellulose extracted from juniper fiber and modified with NaOH	0.26	[68]
	Microfbrillated cellulose modified with aminopropyltriethoxysilane	4.195	[42]
	Treated olive stones	49.3	[69]
	Rice husk modified with epichlohydrin	11.12	[66]

## Conclusion

From the results obtained the following conclusions can be derived.

1. A novel diaminoguanidine-modified cellulose (DiGu.MC), was successfully synthesized and characterized by several characterization techniques including FTIR, SEM and BET.
2. DiGu.MC has proven to be an efficient adsorbent for Cu^2+^, Hg^2+^, Pb^2+^ and Cd^2+^ with adsorption capacities of 66, 55, 70, and 41 mg g^−1^, respectively.
3. Various essential parameters including optimum pH, temperature, contact time, and isotherms were studied and the best condition through which these metal ions can be adsorbed on the surface of the fibers were evaluated.
4. The optimum pH for the uptake of the metal ions was found to be 6 and the process of metal ions uptake by the modified fibers was spontaneous under the different studied temperatures.
5. Adsorption of metals by DiGu-MC is proposed to match pseudo-second order kinetic model and Langmuir isotherm model indicating that the monolayer adsorption of metal ions on the surface of the DiGu.MC adsorbent occurred on homogeneous surfaces and the adsorption mode belonged to chemisorption type.
6. In addition, adsorption process was found to be exothermic and spontaneous at different temperatures based on thermodynamic investigations.
7. The mechanism of metal ions adsorption was the chelation mechanism between N active sites on the DiGu.MC. Based on compositional studies, the DiGu.MC chelating fibers can bind to metal ions C=N, –NH and or –NH_2_ donors forming four and/or five-membered chelate rings.
8. DiGu.MC has excellent reusability results, which make it a unique adsorbent to remediate polluted water from copper, mercury, lead and cadmium ions.

## Supplementary Information

**Additional file 1: Fig. S1. FTIR of **(a)Native cellulose, (b)DAC, (c) DiGu.MC. (d) DiGu.MC-Cu(II)**. Fig. S2** TGA curves of (a) DiGu-MC, (b) Cu-DiGu-MC, (c) Hg-DiGu-MC, (d) Pb-DiGu-MC (e) Cd-DiGu-MC. **Fig. S3.** Effect of initial concentration on adsorption of heavy metals by DiGu-MC. **Table S1**: Specific Surface areas of native cellulose and DiGu-MC fibers.

## Data Availability

All data generated or analysed during this study are included in this published article [and its Additional files].
