# Supplementary material for: Novel diaminoguanidine functionalized cellulose: synthesis, characterization, adsorption characteristics and application for ICP-AES determination of copper(II), mercury(II), lead(II) and cadmium(II) from aqueous solutions
Source: BMC Chem. 2022 Aug 30;16(1):65. doi: 10.1186/s13065-022-00857-3 (PMC9426243; doi:10.1186/s13065-022-00857-3)
Supplement: Supplementary file 1 — Additional file 1: Fig. S1. FTIR of (a)Native cellulose, (b)DAC, (c) DiGu.MC. (d) DiGu.MC-Cu(II). Fig. S2 TGA curves of (a) DiGu-MC, (b) Cu-DiGu-MC, (c) Hg-DiGu-MC, (d) Pb-DiGu-MC (e) Cd-DiGu-MC. Fig. S3. Effect of initial concentration on adsorption of heavy metals by DiGu-MC. Table S1: Specific Surface areas of native cellulose and DiGu-MC fibers. [file 13065_2022_857_MOESM1_ESM.docx]

**SUPPLEMENTARY INFORMATION**

**Novel diaminoguanidine functionalized cellulose: synthesis, characterization, adsorption characteristics and application for ICP-AES determination of copper(II), mercury(II), lead(II) and cadmium(II) from aqueous solutions**

**Magda A Akl^1^*, Mohammed A Hashem^1^, Mohammed A Ismail^1^ and Dina A Abdelgalil^1^**

**^1^**Department of Chemistry, Faculty of Science, Mansoura University, Mansoura 35516, Egypt

*To whom all correspondence should be addressed: Prof Magda Akl

e. mail: magdaakl@yahoo.com

| 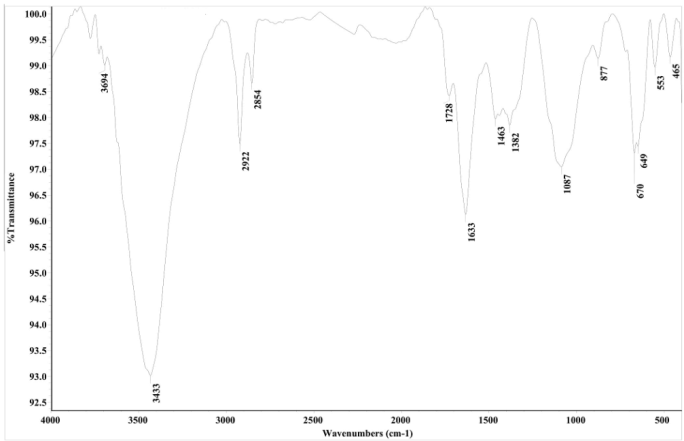  (a)Native cellulose |
| --- |
| 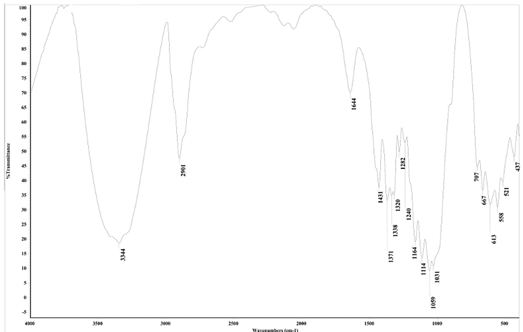  (b)DAC |
| 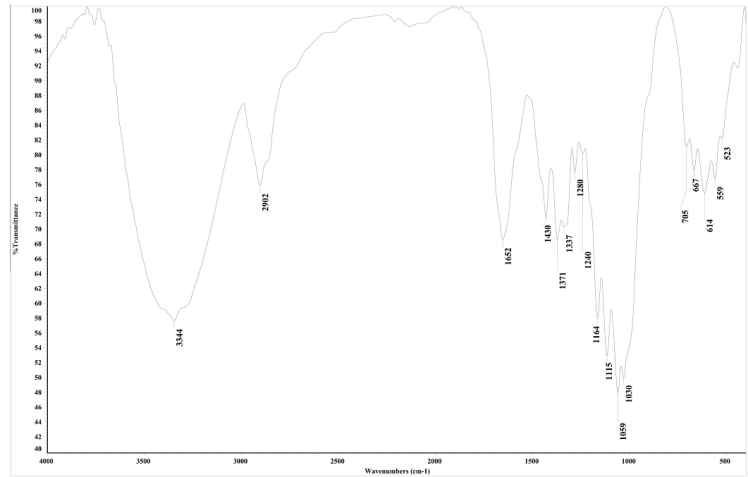  (c) DiGu.MC |
| 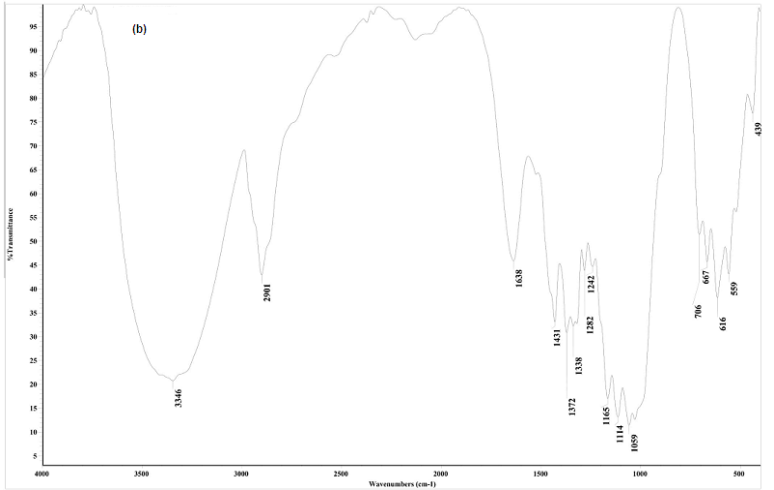  (d) DiGu.MC-Cu(II) |

**Fig. S1. FTIR of** (a)Native cellulose, (b)DAC, (c) DiGu.MC (d) DiGu.MC-Cu(II)


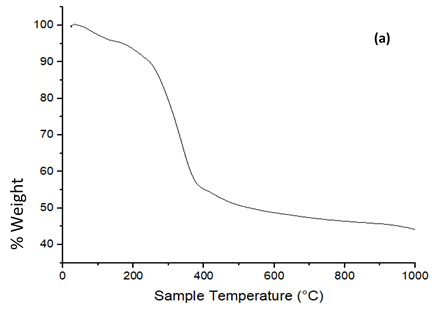

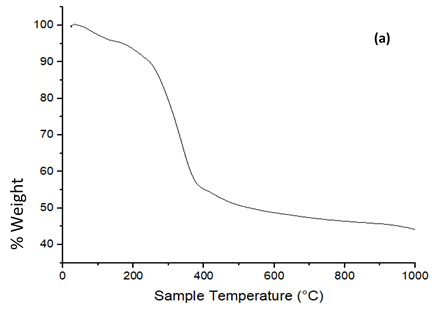

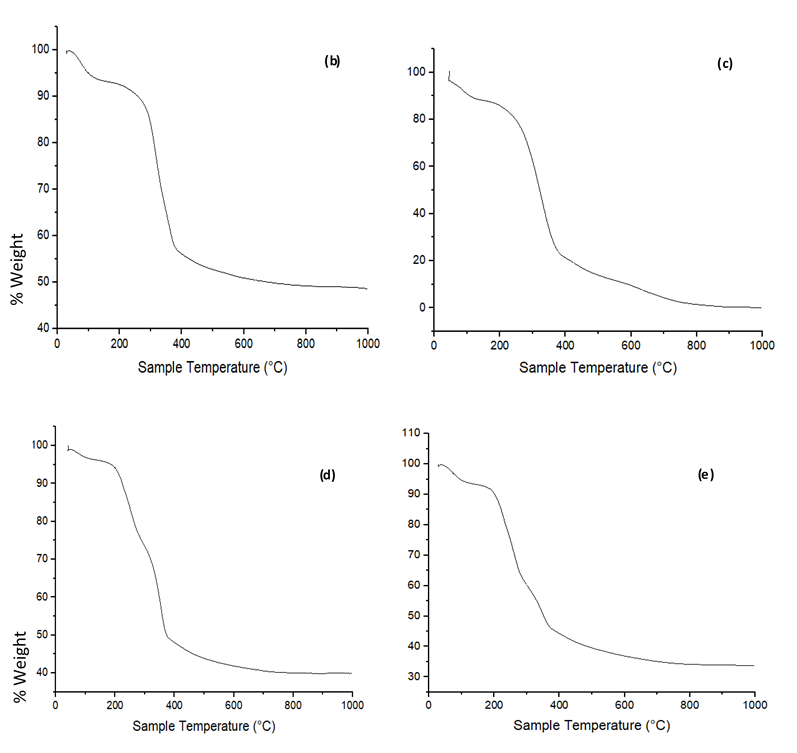

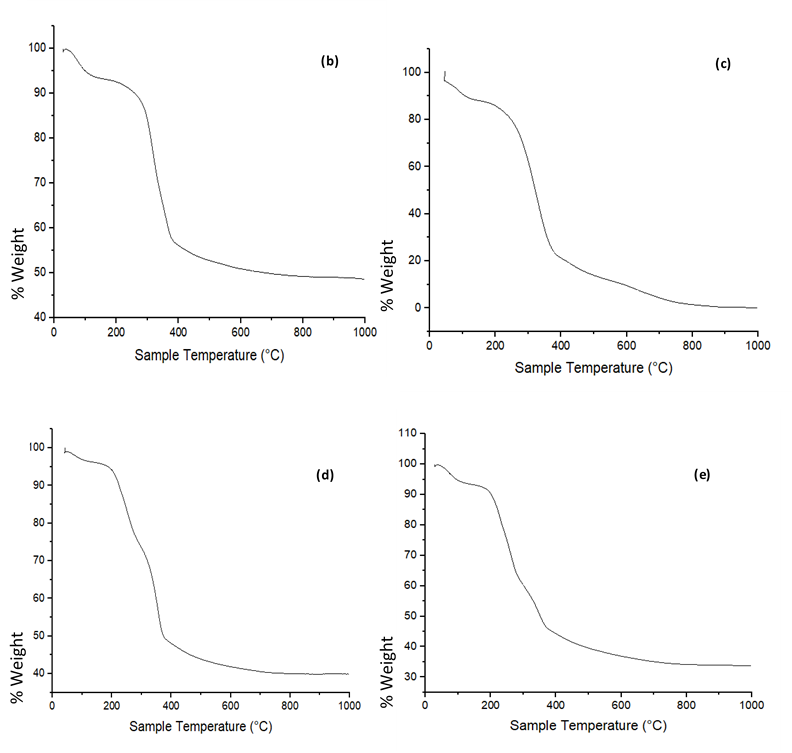

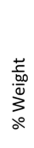

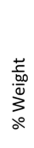

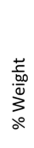


**Fig. S2** TGA curves of (a) DiGu-MC, (b) Cu-DiGu-MC, (c) Hg-DiGu-MC, (d) Pb-DiGu-MC

(e) Cd-DiGu-MC.

**
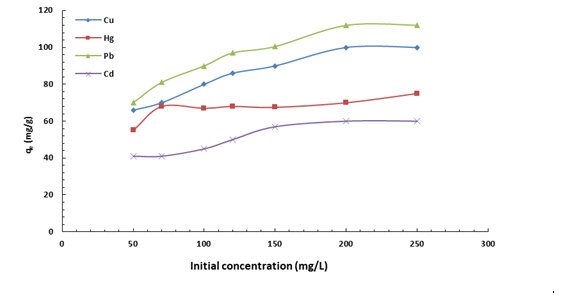
**

**Fig. S3.** Effect of initial concentration on adsorption of heavy metals by DiGu-MC

**Table S1**: Specific Surface areas of native cellulose and DiGu-MC fibers.

| Samples | BET surface area (m^2^ g^−1^) |
| --- | --- |
| Native ellulose | 10.400 |
| DiGu.MC | 3.4028 |
